# Corrigendum

**DOI:** 10.1155/DTE.4.51

**Published:** 1997


					Diagnostic and Therapeutic Endoscopy, Vol. 4, pp. 51
Reprints available directly from the publisher
Photocopying permitted by license only
(C) 1997 OPA (Overseas Publishers Association)
Amsterdam B.V. Published in The Netherlands
by Harwood Academic Publishers
Printed in Singapore
CORRIGENDUM
In the paper entitled "Effect of Diazepam Sedation on Arterial Oxygen Saturation and Patient Tolerance During
Esophagogastroduodenoscopy" by G.M. Malik et al., published in Vol. 3 No. 3, pp. 171-175, there is an error
in the explanation notes to Figure 1. The correction (P > 0.05) is now published below, together with the original
figure.
100
98
97
96
0 95
94
9O
Basal $ 3 5 7 9 11 Recovery
ntubation Extubation
DURATION OF ENDOSCOPIC PROCEDURE (MINUTES)
Control group. (R)Study group. (R)P > 0.05 (Not significant)
FIGURE Time course changes in arterial oxygen saturation (SaO2) during the endoscopic procedure. Endoscopy
induced a transient fall in SaO2 in the first few minutes among both the study and the control groups. However, SaO2continued
to be at a lower level till extubation in the study group (Note: vertical lines represent mean + standard deviation).
51

				

